# Proton pump inhibitors and gastroenteritis

**DOI:** 10.1007/s10654-016-0136-8

**Published:** 2016-03-10

**Authors:** Robert-Jan Hassing, Annelies Verbon, Herman de Visser, Albert Hofman, Bruno H. Stricker

**Affiliations:** 1 Department of Epidemiology, Erasmus Medical Centre, Wytemaweg 80, 3015 CN Rotterdam, The Netherlands; 2 Department of Internal Medicine, Rijnstate Hospital, Arnhem, The Netherlands; 3 Department of Medical Microbiology and Infectious Diseases, Erasmus Medical Centre, Rotterdam, The Netherlands; 4 Star Medisch Diagnostisch Centrum, Rotterdam, The Netherlands; 5 Inspectorate of Health Care, Utrecht, The Netherlands

**Keywords:** Gastroenteritis, Proton pump inhibitor, *Campylobacter*, *Salmonella*, Cohort study

## Abstract

An association between proton pump inhibitor (PPI) therapy and bacterial gastroenteritis has been suggested as well as contradicted. The aim of this study was to examine the association between the use of PPIs and occurrence of bacterial gastroenteritis in the prospective Rotterdam Study. The Rotterdam Study is a population-based cohort study among 14,926 subjects aged 45 years and older with up to 24 years of follow-up. Analyses were performed with a generalized estimating equations method in participants who handed-in a diagnostic stool sample. Furthermore, a nested case–control analysis was performed using the total cohort as a reference group. A bacterial microorganism was isolated in 125 samples, whereas 1174 samples were culture negative. In the generalized estimating equations analysis, we found that participants with a bacterial gastroenteritis were more likely than controls to be current users of PPIs (adjusted OR 1.94; 95 % CI 1.15–3.25). Different sensitivity analyses did not change this result. A considerably higher effect was observed (adjusted OR 6.14; 95 % CI 3.81–9.91), using the total cohort as a reference in a nested case–control analysis. Current PPI therapy is associated with an increased risk of bacterial gastroenteritis. However, by reducing the risk of selection and information bias in our study design, we demonstrated that the effect is lower than previously assumed.

## Introduction

Proton pump inhibitors (PPIs) are among the most frequently prescribed drugs worldwide, but seem to be associated with an increased risk of bacterial gastroenteritis [1–9]. As a result warnings are introduced for people using PPIs, such as avoiding raw meat consumption and antibiotic treatment on demand for travels to the tropics, to prevent food borne infections. This cohort study was designed to asses if the magnitude of this risk, warrants preventive recommendations.

Common indications for PPIs are dyspepsia, peptic ulcer disease, reflux esophagitis, and Barrett’s oesophagus [10]. PPIs reduce gastric acid production by up to 99 % by irreversible blocking of H+/K+ ATPase of parietal cells in the stomach [11]. They have a maximal effect within 4 days and the effect may persist up to 3 days after stopping use [12]. Associations between PPIs and infectious adverse events such as pneumonia, Clostridium difficile-associated diarrhoea and bacterial gastroenteritis have often been described [1–9, 13–17]. An increased risk of gastroenteritis might be explained by the strong reduction in gastric acid resulting in increased susceptibility to bacterial infections. Exogenous bacteria are usually destroyed in the stomach when the pH is <3.0. For species such as *Vibrio cholerae* and *Campylobacter jejuni* it has been shown in vitro that they are very sensitive to pH [18]. However, *Salmonella* species have been found to respond to low pH by developing adaptive mechanisms that allow survival in acid environments [19]. Furthermore, PPIs change the gut flora, which provides a homeostatic protection against ingested pathogens [20, 21]. PPIs also reduce the antibacterial activity of neutrophils which may facilitate *Salmonella* and *Campylobacter* infections [22, 23]. Several case–control studies have shown an increased risk of acquiring gastrointestinal infections caused by *Campylobacter* or *Salmonella* species in patients using PPIs [1–8]. In these case–control studies, a relatively high adjusted odds ratio (aOR) or relative risk was observed, ranging from 2.9 to 11.7. In one nested case–control study, in which participants with a gastroenteritis prior to first PPI prescription were excluded, a considerably lower effect was observed (aOR 1.6) [9]. It has even been stated that there is no evidence that PPIs are associated with gastrointestinal infections based on outcomes adjusted for pre-treatment susceptibility to bacterial gastrointestinal infections and time-dependent confounding factors [24], which observation suggests that previous case–control studies have suffered from selection or information bias. Therefore, we designed a nested case control study within The Rotterdam Cohort, a prospective cohort study, to examine the association between the use of PPIs and occurrence of bacterial gastroenteritis. To minimize the risk of information bias we used participants with negative stool samples as a control group. To test the hypothesis that an incorrect control group will influence the study results we also analysed the association using the total cohort as a control group.

## Materials and methods

### Study population

The study was performed in The Rotterdam Study, a prospective population-based cohort study in 14,926 people aged ≥45 years, from one district (Ommoord) in the city of Rotterdam, the Netherlands [25]. In short, from 1990 through 1993, 7983 participants were included (cohort I). In 2000 an additional 3011 participants who had become 55 years old or older or who had moved into the district, were enrolled (cohort II). In 2006 another 3932 participants, aged 45 years and older were included (cohort III). Follow-up examinations are conducted every 4–5 years. Participants are continuously monitored through linkage of records from general practitioners.

The Rotterdam Study was approved by the medical ethics committee according to the Wet Bevolkingsonderzoek ERGO (Population Study Act Rotterdam Study) executed by the Ministry of Health, Welfare and Sports of the Netherlands. All study participants provided written informed consent.

### Definition of outcome

A case was defined as a community-dwelling non-hospitalized individual with a positive stool sample for *Campylobacter*, S*almonella*, *Yersinia* or *Shigella* species. A control was defined as an individual with a negative stool sample. Stool sample results were obtained from Star Medisch Diagnostisch Centrum (Star-MDC), a centre for medical diagnostics for outpatients in the city of Rotterdam. The majority of all laboratory tests, including microbiology tests, of patients from general practitioners within the Ommoord district of Rotterdam are performed at Star-MDC. Of all participants of The Rotterdam Study, of whom informed consent was obtained for requesting medical information, positive and negative microbiology tests between 1999 and April 2013 were obtained. Stool samples were selected and samples in which parasites were isolated were excluded. Detection of bacterial enteric pathogens in stool samples at Star-MDC is performed by Multiplex polymerase chain reaction (PCR), followed by culture and microscopy in case of a positive result. Until December 2010, when PCR was introduced at Star-MDC, detection of bacterial enteric pathogens was performed by conventional culture and microscopy only.

### Assessment of exposure and covariables

Participants were considered as current user of PPI if the calendar date of the stool sample fell within a prescription episode of a PPI. Prescription episodes were calculated by dividing the total number of supplied pills by the recommended daily number. Additional covariables assessed were age, sex, cohort, calendar date (year), BMI, household status, past use of proton pump inhibitors, current or past use of H2-receptor antagonists, current use of chronic medication (antidiabetic medication, antihypertensive medication, or statins), intestinal anti-inflammatory agents, corticosteroids, immunosuppressant medication, meat consumption, red meat consumption, chicken consumption, egg consumption and alcohol consumption (for all dietary variables: yes/no and gram/days).

BMI and household status was obtained from baseline characteristics of The Rotterdam Study. Medication use was obtained through automated linkage with pharmacy filled prescription data, available from January 1st, 1991 until April 2013. Dietary data were available from 1 week food consumption questionnaires obtained at the first visit of cohort I and cohort III and at the third visit of cohort II. Multiple imputation (10×) of missing dietary data and BMI was performed using all covariables.

### Statistical analyses

#### Model design

To assess the association between current use of PPIs and gastrointestinal infections generalized estimating equations (GEE) was used to adjust for correlation between repeated measurements in the same participants, using a varying between-measurement time window [26, 27]. The working correlation matrix in which the model had the lowest quasi likelihood under independence model criterion was selected. The model was adjusted for age and sex and additionally for covariables changing the point estimate (β) of current PPI use by more than 10 % or if considered clinically relevant.

#### Sensitivity analyses

Different sensitivity analyses were performed. To exclude confounding by indication or protopathic bias we recoded PPI use started within 14 days before a positive stool sample as non-use. To take into account that in many occasions PPIs are not used on a daily basis, and therefore the actual period of exposure will probably exceed the period calculated based on the pharmacy data, we also included use during the past 14 and 30 days as potentially exposed cases in sensitivity analyses. To exclude confounding by contra-indication we censored every participant at the first case in a sensitivity analysis. Participants receiving medication from other sources than the pharmacy, such as nursing home residents, might introduce information bias. Therefore we did a sensitivity analysis excluding participants without pharmacy prescriptions during the last 90 days because nursing home residents do not obtain medication through a community pharmacy. Furthermore, we performed a sensitivity analysis for *Campylobacter* species and *Campylobacter* or *Salmonella* species only. Different analyses were stratified on sex.

#### Additional analysis

We also analysed the exposure of PPIs in a nested case–control analysis to assess the association between PPIs and gastrointestinal infections in the total population of The Rotterdam Study. The use of PPIs was used as a time-varying determinant of exposure as previously described [28]. To use the total population of The Rotterdam Study every case (participant with a positive stool sample) was matched to every other participant alive and eligible at the same calendar date (day). Each time a case was identified, exposure in this case was compared with exposure in the other participants (cases might become controls, and controls might become cases). The model was adjusted for age and sex and additionally for covariables used in the final model.

A two-sided *p* value below 0.05 was considered statistically significant. Statistical analyses were performed using SPSS version 21.0 (IBM Corp., Somers, NY, USA).

## Results

During the study period, 1329 stool cultures of participants of The Rotterdam Study were identified at Star-MDC. We excluded 30 stool samples because of isolation of a parasite, resulting in 1299 stool samples for the study. A bacterial microorganism was isolated in 125 samples, whereas 1174 samples were culture negative. In total 105 (84.0 %) *Campylobacter* species, 16 (12.8 %) *Salmonella* species, 3 (2.4 %) *Yersinia* species and 1 (0.8 %) *Shigella* sonnei were isolated. All 125 positive stool samples were collected from 118 different participants and all 1174 negative stool samples from 903 different participants. The percentage of missing data of BMI was 7.6 % and of dietary data 28.6 %. Characteristics of cases and controls are shown in Table 1.

**Table 1:** Baseline characteristics of participants with stool sample

	Participants with positive stool sample	Participants with negative stool sample
Total	118*	903
Age—year (SD)	65.1 ± 10.3	68.1 ± 12.8
Male sex—no. (%)	49 (41.5)	301 (33.3)
*Cohort (%)*		
I	34 (28.8)	325 (36.0)
II	35 (29.7)	227 (25.1)
III	49 (41.5)	351 (38.9)
Household alone—no. (%)	20 (16.9)	229 (25.4)
BMI^‡^ (SD)	25.2 ± 4.2	24.5 ± 4.3
*Use of proton pump inhibitors—no. (%)*	43 (36.4)	242 (26.8)
Current use for 14 days or more	32 (27.1)	152 (16.8)
Including use during past 14 days	50 (42.4)	269 (29.8)
Including use during past 30 days	55 (46.6)	288 (31.9)
>1 Defined daily dose/day	13 (30.2)	76 (31.4)
Past use only	33 (28.0)	289 (32.0)
*Medication use—no. (%)*		
H2-receptor antagonists	3 (2.5)	22 (2.4)
H2-receptor antagonists—past use	37 (31.4)	312 (34.6)
Antidiabetic medication	12 (10.2)	93 (10.3)
Antihypertensive medication	50 (42.4)	316 (35.0)
Statins	27 (22.9)	151 (16.7)
Antidiabetic, antihypertensive medication or statins	55 (46.6)	376 (41.6)
Intestinal anti-inflammatory agents	1 (0.8)	4 (0.4)
Corticosteroids	3 (2.5)	15 (1.7)
Immunosuppressant medication	0	6 (0.7)
*Dietary—no. (%)*		
No meat consumer^†^	2 (2.4)	6 (0.7)
No red meat consumer^∏^	2 (2.4)	11 (1.2)
No chicken consumer^∏^	15 (18.3)	105 (11.6)
No egg consumer^∏^	5 (6.1)	48 (5.3)
Alcohol^$^	72 (61.0)	558 (61.8)
*Dietary—gram/days (SD)*		
Meat^†^	110.9 ± 60.4	103.3 ± 54.4
Red meat^∏^	91.7 ± 55.0	85.5 ± 50.0
Chicken^∏^	17.1 ± 18.1	16.4 ± 16.5
Eggs^∏^	15.0 ± 11.1	15.7 ± 12.9
Alcohol^$^	13.0 ± 15.7	13.4 ± 15.2

* Out of total 125 positive isolates

^†^Patients with positive stool sample *N* = 82, negative stool sample *N* = 644

^‡^Patients with positive stool sample *N* = 111, negative stool sample *N* = 839

^∏^Patients with positive stool sample *N* = 82, negative stool sample *N* = 646

^$^Patients with positive stool sample *N* = 93, negative stool sample *N* = 712

### Model design

For the generalized estimating equations, the independent working correlation structure had the best fit. Designing the final model, past use of H2-receptor antagonists increased the point estimate (β) of current PPI use, adjusted for age and sex, by more than 10 %, whereas current use of chronic medication, all decreased the effect by more than 10 %. We included age, sex, cohort, calendar date, past use of proton pump inhibitors, past use of H2-receptor antagonists, and current use of chronic medication in the final model (model 2). In the final model we found that participants with a bacterial gastroenteritis were more likely than control participants to be current users of PPIs, with an aOR of 1.94 (95 % CI 1.15–3.25) (Table 2).

**Table 2:** Association of use of proton pump inhibitors with bacterial gastrointestinal infections

	Number of cases	Number in cohort	Use of PPI (%)	Cases *N* = 125 OR (95 % CI)	*P* value
*Current use of proton pump inhibitor*					
Univariate analysis	125	1299	375 (28.9)	1.50 (1.01; 2.23)	0.047
Adjusted analysis—model 1	125	1299	375 (28.9)	1.62 (1.09; 2.43)	0.018
Adjusted analysis—model 2	125	1299	375 (28.9)	1.94 (1.15; 3.25)	0.013
*Sensitivity analyses*—*model 2*					
Only including current use for 14 days or more	125	1299	242 (18.2)	1.99 (1.19; 3.35)	0.009
Including use during past 14 days	125	1299	422 (32.5)	2.14 (1.35; 3.38)	0.001
Including use during past 30 days	125	1299	453 (34.9)	2.28 (1.46; 3.55)	<0.001
Censored at first case	118	1246	353 (28.3)	2.02 (1.19; 3.42)	0.009
Excluding no medication for >90 days	124	1223	375 (30.7)	1.78 (1.05; 3.01)	0.032
*Campylobacter* only	105	1279	368 (28.8)	1.93 (1.11; 3.36)	0.019
*Campylobacter* and *Salmonella*	121	1295	375 (29.0)	2.05 (1.20; 3.49)	0.008
Male only	53	436	131 (30.0)	3.28 (1.44; 7.49)	0.005
Female only	72	863	244 (28.3)	1.31 (0.66; 2.60)	0.45

Generalized estimating equations method, negative stool cultures as control group

Model 1: adjusted for sex, age

Model 2: adjusted for sex, age, cohort, calendar date, past use of proton pump inhibitors, current use of chronic medication, past use of H2-receptor antagonists

### Sensitivity analyses

Different sensitivity analyses did not result in a significant change of the effect (Table 2). A sensitivity analysis including use during the past 14 and 30 days resulted in aORs of 2.14 (95 % CI 1.35–3.38) and 2.28 (95 % CI 1.46–3.55), respectively. Excluding current use of PPIs for 14 days or more, to exclude confounding by indication or protopathic bias, resulted in an aOR of 1.99 (95 % CI 1.19–3.35). Censoring at the first case, to exclude confounding by contra-indication, resulted in an aOR of 2.02 (95 % CI 1.19–3.42) and excluding participants without prescriptions during the last 90 days in an aOR of 1.78 (95 % CI 1.05–3.01). An aOR of 1.93 (95 % CI 1.11–3.36) and 2.05 (95 % CI 1.20–3.49) was observed in sensitivity analyses for including only *Campylobacter* and *Campylobacter* or *Salmonella*, respectively (Table 2). After stratifying on sex a difference was observed between male (aOR 3.28; 95 % CI 1.44–7.49) and female (aOR 1.31; 95 % CI 0.66; 2.60) participants (Table 2).

### Additional analysis

In a matched case–control analysis, adjusting for the same covariables of the final model, but using all other participants of The Rotterdam Study as control group, a considerably higher aOR of 6.14 (95 % CI 3.81–9.91) was observed compared to the GEE analysis using negative stool samples as control group (Table 3).

**Table 3:** Association of use of proton pump inhibitors with bacterial gastrointestinal infections

	Number of cases	Number in cohort	Use of PPI (%)	Infections *N* = 125 OR (95 % CI)	*P* value
			Total cohort *N* = 12,515		
*Current use of proton pump inhibitor*					
Univariate analysis	125	12,515	11.7*	3.35 (2.31; 4.87)	<0.001
Adjusted analysis—model 1	125	12,515	11.7*	4.03 (2.77; 5.87)	<0.001
Adjusted analysis—model 2	125	12,515	11.7*	6.14 (3.81; 9.91)	<0.001
Male only—model 2	53	5,113	10.2^†^	11.07 (5.51; 22.24)	<0.001
Female only—model 2	72	7,402	12.7^‡^	3.83 (1.96; 7.47)	<0.001

Nested case–control analysis—all other participants of The Rotterdam Study as control group

Model 1: adjusted for sex, age

Model 2: adjusted for sex, age, cohort, calendar date, past use of proton pump inhibitors, current use of chronic medication, past use of H2-receptor antagonists

* Percentage over 125 strata

^†^Percentage over 53 strata

^‡^Percentage over 72 strata

## Discussion

PPIs have been associated with an increased risk of bacterial gastroenteritis in previous studies [1–9]. In this population based cohort study we found that current PPI therapy was associated with a strongly increased risk of bacterial gastroenteritis, with an aOR of more than six. However, this risk decreased to 1.94 after restriction to the subgroup with stool samples. Therefore, we suspect that information bias inflated the risks in other population based studies which have shown an association between PPI therapy and an increased risk of *Campylobacter* and *Salmonella* infections before. Although, people using PPIs still should be careful consuming food which could be contaminated, such as beef, poultry or processed food, the risk of gastroenteritis is probably less than previously assumed.

One of the strengths of our study is that we tried to use a comparable control group by only using participants with negative stool samples. In most of the previous population based studies the magnitude of the effect may have been overestimated as a result of the use of incomparable control groups. Typically, case control studies regarding use of PPIs may suffer from a “healthy control” bias. We further tried to avoid healthy control bias by correcting for the use of chronic medication. Some of the previous studies used a random sample of population registries, or volunteering friends or relatives of the cases as control group [2, 4–6]. These participants are probably healthier and will therefore differ considerably from the cases. But also commonly used strategies, such as using matched controls, obtained from general practitioner databases, will not prevent information bias [1]. Studies considering self-limiting diseases such as gastroenteritis, might select a “help seeking” and therefore biased population. As a consequence, people using a PPI will be more inclined to consult medical help in case of gastroenteritis compared to randomly selected controls, even if they are matched.

Residual confounding might also have influenced previous study results, because dietary pattern, which has been shown to be the most important risk factors for *Campylobacter* and *Salmonella* infections has not been included in these studies [29, 30]. An association with dietary data was not observed in our study. Unfortunately, the number of missing dietary data in our study was rather high with as a consequence a large number of imputed data.

Observational studies may always suffer from bias and residual confounding. We had no data on other important risk factors for bacterial gastroenteritis, such as foreign travelling, eating in a restaurant, or contact with animals [29, 30].

We believe we used a comparable control group using participants with negative stool samples. However, results from a test-negative study design may also underestimate the risk, since a number of the controls with negative stool cultures may be false-negatives. They can be false-negative either because of low diagnostic sensitivity against the four bacterial species (e.g., too little material received, long transportation time, stool collected many days after onset of gastroenteritis) or because the patient suffered from other causes of bacterial gastroenteritis (diarrheagenic *Escherichia coli* or *Clostridium difficile*).

Furthermore, in elderly gastric acid secretion is impaired compared to younger aged individuals [31]. If gastric acid secretion is already impaired, PPIs will have a smaller effect. Therefore, the smaller risk estimate in our study might be explained by the fact that our population mainly consisted of elderly, in which gastric acid secretion is already impaired.

Because *Salmonella* species are less susceptible to pH, the association between PPI therapy and gastroenteritis might be smaller for *Salmonella* species [19]. Unfortunately, however, the number of *Salmonella* infections in this study was too small to draw conclusions on this association.

During the study period the method of detection of enteric pathogens changed by the introduction of PCR, resulting in an increased sensitivity. To correct for an increased risk of false negative stool samples in the earlier years of the study, we included calendar date and cohort in the assessment of model design.

Although male gender has been shown to be an independent risk factor for bacterial gastroenteritis, it has not been shown before that the association between PPI therapy and bacterial gastroenteritis is much higher for males [32]. We were unable to explain this result by other covariables. Possibly there are unmeasured (hygiene related) behavioural aspects to explain this difference. In experimental studies a gender difference was observed between male and female neutrophils. Male neutrophils show higher responsiveness to stimulation with lipopolysaccharide and interferon-ϒ [33]. Neutrophils play an important role in *Salmonella* and *Campylobacter* infections [22, 23]. The harmful effect of PPIs may therefore be greater for male than for female subjects. Of course, studies are needed to test this rather speculative hypothesis.

In conclusion, current PPI therapy was associated with an increased risk of bacterial gastroenteritis. We demonstrated that the effect is lower than previously assumed, by reducing the risk of information bias in our study design.
